# Resolving ecological drivers of temporal variations of β-diversity across intertidal microbiomes

**DOI:** 10.1093/ismeco/ycaf025

**Published:** 2025-02-17

**Authors:** Xia Liu, Xiaofan Gong, Kai Ma, Wen Song, Jiayin Zhou, Mengqi Wang, Yueyue Li, Mengzhi Ji, Yan Li, He Han, Yuzhuo Wang, Qichao Tu

**Affiliations:** Institute of Marine Science and Technology, Shandong University, Qingdao, Shandong 266237, China; Institute of Marine Science and Technology, Shandong University, Qingdao, Shandong 266237, China; Institute of Marine Science and Technology, Shandong University, Qingdao, Shandong 266237, China; Institute of Marine Science and Technology, Shandong University, Qingdao, Shandong 266237, China; Institute of Marine Science and Technology, Shandong University, Qingdao, Shandong 266237, China; Institute of Marine Science and Technology, Shandong University, Qingdao, Shandong 266237, China; Institute of Marine Science and Technology, Shandong University, Qingdao, Shandong 266237, China; Institute of Marine Science and Technology, Shandong University, Qingdao, Shandong 266237, China; Institute of Marine Science and Technology, Shandong University, Qingdao, Shandong 266237, China; Institute of Marine Science and Technology, Shandong University, Qingdao, Shandong 266237, China; Institute of Marine Science and Technology, Shandong University, Qingdao, Shandong 266237, China; Institute of Marine Science and Technology, Shandong University, Qingdao, Shandong 266237, China; Qingdao Key Laboratory of Ocean Carbon Sequestration and Negative Emission Technology, Shandong University, Qingdao, Shandong 266237, China

**Keywords:** β-diversity, temporal variation, species pool, community assembly, environmental heterogeneity

## Abstract

Resolving the ecological drivers mediating the diversity patterns of microbial communities across space and through time is a central issue in microbial ecology. Both regional species pools and local community assembly contribute to the spatial turnover of biodiversity. In this study, we extended the concept of regional species pool to temporal, and investigated the seasonal dynamics of intertidal microbiomes across four microbial domains/kingdoms (bacteria, archaea, fungi, and protists). The results showed that the seasonal variations of microbial β-diversity were primarily governed by community assembly processes rather than temporal species pools. Different microbial domains/kingdoms were structured by different ecological processes, with homogeneous selection as the major process for all of them. Additionally, bacteria and fungi were critically shaped by drift, and protists by drift and homogeneous dispersal. Among various factors, temperature was important in shaping the temporal patterns of microbial β-diversity. The fluctuation in temperature was strongly associated with fungi and protists, resulting in high drift of community composition. This study demonstrated that community assembly processes governed the dynamic seasonal β-variations of intertidal microbiomes, expanding our understanding from spatial ecology.

## Introduction

A central question in ecology is to uncover the ecological drivers mediating the patterns of biological communities across space and through time. Both macrobial and microbial communities in nature follow several well recognized ecological patterns such as SAD (species abundance distribution) [1–4], LDG (latitudinal diversity gradient) [5–7] and DDR (distance decay relationship) [8, 9], but the forms and strength could be substantially different owing to their distinct biophysiochemical properties [10, 11]. For instance, macroorganisms, such as plants and animals, typically exhibit pronounced distance decay relationships at larger spatial scales due to their limited dispersal capacity and significant impact of environmental heterogeneity, including variations in climate and soil type, on their distributions [12]. In contrast, distance decay relationships in microbial communities can occur significantly at much smaller spatial scales (even on the scale of a few meters to a few kilometers) [13–15]. However, due to the highly dispersive nature of microorganisms, distance decay tends to manifest more slowly at large scales. This suggests that microorganisms are capable of widespread dispersal through a variety of mechanisms (e.g. air currents, water currents, animals, etc.), thereby slowing the rate of decline in similarity of community composition with increasing geographic distance [12, 15–17].

Variations in β-diversity across latitudinal gradients have garnered attention, reflecting differences among local communities influenced by local (α-diversity) and regional diversity (γ-diversity) [18–22]. Notably, contrasting mechanisms drive β-diversity in microbial and macrobial communities: regional species pools impact plant communities across latitudinal and elevational gradients, while local community assembly is crucial for microbial communities along latitudes [19, 20, 23]. Recent studies highlight that sampling scale significantly affects community variation drivers [14, 20, 24, 25]. At a large scale, regional species pools are influenced by environmental gradients, species migration, and geographic isolation, which can reduce β-diversity [18, 26–28]. Conversely, at smaller scales, local habitat conditions, species competition, and interactions play key roles in shaping β-diversity [23, 29–31].

In parallel with spatial patterns, the temporal variation of biological communities is another important topic in ecology, especially for predicting future scenarios [32–34]. Compared to spatial β-diversity variations, much less effort has been made to investigate the variations of temporal composition of microbial communities [35–37]. The temporal variations of biological communities can be driven by a multitude of factors [38], including environmental changes [39], ecological interactions [40], and stochastic processes [41, 42]. For instance, seasonal changes in temperature, precipitation, and resource availability can influence the abundance and distribution of species within a community. Similar to spatial patterns, biological processes such as reproduction, dispersal, and mortality also contribute to temporal variations by affecting population dynamics over time [34, 36, 43]. Owing to the critical ecosystem functions that microbes play, elucidating the temporal dynamics of microbial communities is essential for predicting ecosystem responses to environmental changes and efficient ecosystem service management.

Community composition of micro- and macro-organisms at a given location and time results from multiple ecological processes, including selection, dispersal, drift, and speciation [44–46]. However, many previous metacommunity studies mainly capture spatial patterns, overlooking the temporal dynamics of microbial communities [44, 45, 47]. Recently, effort have been made to explore the temporal dynamics of microbial communities in different ecosystems, including those in the human gut [48, 49], soil [50, 51], and aquatic environments [52, 53]. Understanding these patterns is crucial for grasping biodiversity maintenance, ecosystem functioning, and community responses to environmental changes [54–56]. For example, studies on soil microbial eukaryotic communities have documented significant seasonal variations [57, 58]. These findings are mirrored in investigations of microbial assembly responses, revealing how environmental changes, including disturbances and fluctuations, influence community structures. Microbial communities can exhibit distinct assembly processes over time when being subjected to various environmental changes, including disturbances and periodic or stochastic fluctuations.

Intertidal ecosystems serve as the dynamic interfaces between land and sea, enduring frequent changes in temperature, salinity, and oxygen due to tidal shifts [59, 60]. The intertidal microbial communities are uniquely adapted to these fluctuations, which drive biodiversity and nutrient cycling by linking terrestrial and marine inputs [61]. The cycles of exposure and submersion challenges and shapes the community structure, highlighting the resilience and ecological significance of intertidal microbes. In this study, aiming to investigate the underlying mechanisms driving the temporal patterns across microbial domains, a bimonthly sampling campaign was conducted in a typical mudflat intertidal area in the Jimo coastal district, Qingdao, China. The following ecological questions were addressed: (1) How do the temporal patterns of intertidal microbial communities differ across different domains? (2) What factors drive the seasonal compositional variations of intertidal microbial communities? (3) How important are temporal species pools compared to community assembly processes in driving seasonal dynamics of intertidal microbial β-diversity? The results revealed distinct temporal patterns across different intertidal microbial domains. Comparatively, community assembly mechanisms played crucial roles in driving the seasonal dynamic changes of β-diversity of intertidal microbiomes. These findings provide important insights into the temporal variations of the complex microbial communities in natural ecosystems.

## Materials and methods

### Experimental design and sample collection

Samples were collected bimonthly from June 2020 to June 2021 in the mudflat intertides near Niedao Harbor (120.75°E, 36.46°N), a typical quagmire intertidal zone located in Jimo District, Qingdao, China (Supplementary Fig. 1). The sampling area spanned ~1.5 km^2^ (750 m in width, 2000 m in length), which was exposed to the air at the low tide level. Samples were collected to cover the whole sampling area as much as possible, aiming to capture the ecological variability across the study area. Specifically, samples were collected at ~500-meter intervals along the length (toward the sea), and at ~250-meter intervals along the width. To ensure consistency and comparability across different time points, sediment samples were collected within the same spatial extent using the same sampling strategy. For each sampling time point, 12 to 15 samples were collected. For each sampling point, a “five-point sampling method” was used, via which five sediment cores were collected and homogenized as one sample. The collected sediment samples (~200 g each) were placed on ice and delivered to the laboratory as soon as the sampling was finished. About 100 g of each sample was freeze-dried for DNA extraction and environmental parameter analysis, while the remaining were stored at −80°C for future use.

### Environmental variable measurements

A total of 11 environmental factors were measured, including temperature, salinity, pH, ammonia nitrogen (NH+ 4-N), nitrate nitrogen (NO-3-N), nitrite nitrogen (NO-2-N), total nitrogen, total phosphorus (TP), total organic carbon (TOC), and sulfate (SO2–4). Temperature was recorded with an infrared thermometer at the sampling time, salinity with a salinometer in a soil-water solution (1:2, w/v) (WS-31, Xudu, Beijing, China), and pH with a pH electrode in a soil-water mixture (1:5, w/v) (STARTER 300, OHAUS, Beijing, China). Total nitrogen was analyzed using Kjeldahl method. TP was determined by ammonium molybdate spectrophotometry (GB/T 11893–89) at 660 nm [62]. TOC was measured using the dichromate digestion method. NH+ 4-N, NO-3-N, and NO-2-N were determined by automated discrete analysis (CleverChem 380) [63]. All environmental parameters were standardized in R using the "scale" function.

### DNA extraction, PCR amplification, and sequencing

Total DNA was extracted from 0.50 g of sediment sample using the FastDNA SPIN kits (MP Biomedicals, USA) according to the manufacturer's protocol. DNA quality and quantity were assessed using a NanoDrop ND-250 spectrophotometer based on the ratios of 260/280 nm and 260/230 nm, respectively. The 16S rRNA V4 region of bacteria, the 16S rRNA V4-V5 region of archaea, the internal transcriptional spacer region (ITS) of fungi and the 18S rRNA V4 region of protist were targeted to analyze different microbial domains/kingdoms by high-throughput sequencing method. The corresponding primers are 338F/806R, 524F-10-ext/Arch958Rmod, gITS-7F/ITS-4R, and 18S-528F/18S-706R, respectively (Supplementary Table 1). The PCR reaction conditions were set to predenaturation at 95°Cfor 5 min, followed by 25 cycles: denaturation at 95°C for 30 s, annealing at 53°Cfor 1 min, extension at 72°Cfor 1 min, and finally extension at 72°C for 10 min. All PCR tests used deionized water as a negative control. Finally, the purified PCR products were mixed with sequencing primers for end-to-end sequencing on the Illumina Miseq PE 250 × 2 sequencer (Illumina Inc., San Diego, USA).

### Experimental data processing

A series of standardized noise reduction processes, including demultiplexing, quality filtering and chimera removal, were performed on the raw data through the “DADA2” [64] package in R v4.2.2. For 16S rRNA and 18S rRNA genes, amplicon sequencing variants (ASVs) obtained by DADA2 pipeline were directly used for subsequent analysis. The PR2 database [65] was used for taxonomic assignment of 18S rRNA gene ASVs. The 16S rRNA gene ASVs were classified by the Ribosomal Database Project (RDP) classifier [66]. For Archaea, only ASVs annotated as Archaea at the domain level were retained, while ASVs annotated as Bacteria were excluded from further analyses. For fungi, Due to the existence of non-fungal eukaryotic ASVs, we first searched ASVs against NCBI database by BLAST (Basic Local Alignment Search Tool), and used MEGAN software to identify non-fungal ASVs. Then, the UNITE database [67] was used to classify the retained fungi ASVs. Due to the variation in sequencing depth of the data in different samples, all samples were randomly subsampled to the same sequencing depth using the "rarefy" function in the "GuniFrac" R package before further statistical analysis.

### Exploring the temporal diversity patterns of intertidal microbial communities

The seasonal turnover of β-diversity from June 2020 to June 2021 were investigated for four intertidal microbial domains/kingdoms. The β-diversity was calculated using the Bray–Curtis dissimilarity index, which measures the dissimilarity in microbial community composition among different samples. This computation was conducted using the 'vegdist' function in the R package “vegan”. We calculated α-diversity using the 'HillR' R package, which allows for the estimation of diversity across multiple orders, denoted by the diversity parameter “q”. The Hill numbers at different orders (q = 0, 1, 2) [68] were computed, respectively representing species richness, Shannon diversity, and Simpson diversity. The Pielou evenness index was computed using the 'diversity' function within the 'vegan' package in R, providing a measure of how evenly individuals are distributed within each community.

Taxa-time relationship (TTR) is a fundamental concept in ecology, aiming to understand how species richness changes over time within specific ecological contexts. It is quantified using the following equation, 


$$ S=c{T}^w $$

where *S* represents observed species richness, *T* denotes the time interval, c is a constant, and *w* signifies the rate of change in species richness over time.

In addition to TTR, the time-decay relationships (TDR), which describes the decline in community similarity within an ecosystem over time, was also analyzed. Here, TDR was quantified using the formula, 


$$ {\log}_{10}\left({S}_S\right)=\mathrm{constant}-w{\log}_{10}(T) $$

where *S_S_* represents pairwise community compositional similarity, *T* denotes the time interval, and *w* is the species turnover rate. Regression analysis is performed to examine this relationship, with logarithmic transformation often used to capture the decay pattern.

Principal Coordinate Analysis (PCoA) was employed to visualize the differences in microbial community composition among different sampling months, based on the Bray–Curtis similarity matrix. Additionally, the Mantel test was utilized to assess correlations between environmental factors and the intertidal microbial communities. These analyses were conducted using the R package “vegan”.

### Disentangling the ecological processes mediating community variations

Our study primarily focused on investigating the impact of temporal species pool and community assembly mechanisms on the seasonal turnover of β-diversity for different intertidal microbial domains. In our research, the species pool is defined as the number and abundance of species observed at each sampling time point, and the species pool size (equivalent to γ-diversity) is defined as the total ASV richness at each time point [69]. To investigate the relationship between species pool size (γ-diversity) and temporal turnover of microbial β-diversity, random sampling without any community assembly processes were initially conducted. The expected relationship between β-diversity and species pool size (γ-diversity) was assessed following Kraft et al [18], assuming the species distribution within the species pool follows a log-normal distribution. If the observed relationship between β-diversity and γ-diversity aligns with the expected pattern and proves to be statistically significant, it underscores the crucial role of the regional species pool in influencing β-diversity variation. Conversely, the absence of a significant correlation between observed β-diversity and γ-diversity suggests that the regional species pool has limited influence on β-diversity variation along the temporal gradient.

To investigate the impact of community assembly processes on seasonal changes in β-diversity after correcting for species pool size (γ-diversity), we employed the "tNST" function in the "NST" R package to generate a null distribution of expected differences through 999 bootstrap iterations. The observed and expected β-diversity were calculated based on Bray–Curtis dissimilarity indices. The β-deviation was determined by the disparity between the observed and expected β-diversity under null models. If the observed β-diversity was infinitely close to the expected β-diversity, then the β-deviation behaved infinitely close to the zero value, suggesting that stochastic processes may dominate the variations of microbial community β-diversity. If the observed β-diversity was consistently greater than the expected β-diversity, the value of β-deviation was consistently greater than zero, suggesting that heterogeneous selection or dispersal limitation was dominant during the seasonal turnover of microbial β-diversity. In contrast, if the observed β-diversity was consistently smaller than the expected β-diversity, the value of β-deviation was negative, meaning that the variations of β-diversity might be mainly influenced by homogeneous selection or homogeneous dispersal.

To further elucidate the significance of community assembly mechanisms in shaping intertidal microbial community composition, the relative contributions of various ecological processes were quantified. By employing the iCAMP [70] approach, we assessed the roles of heterogeneous selection, homogeneous selection, dispersal limitation, heterogeneous dispersal, and drift. This analysis utilized Bray-Curtis-based Raup-Crick and β-Nearest Taxonomic Unit Index metrics. Additionally, we investigated the relationships between observed β-diversity and the community assembly process, as indicated by β-deviation.

## Results

### Seasonal variations of microbial diversity and composition

A total of 99 samples were collected during the sampling course covering seven time points at bimonthly intervals. Through high-throughput amplicon sequencing and followed by quality filtering, chimera removal and clustering, a total of 43 606 bacterial ASVs, 8345 archaeal ASVs, 17 580 fungal ASVs, and 12 664 protist ASVs were obtained. For more accurate data analyses across different samples, the number of sequences per sample were rarefied to 31 722, 35 534, 16 859, and 39 306 for bacteria, archaea, fungi, and protists, respectively. At the phylum level, dominant microbial phyla clearly fluctuated at different time point. Among these, Proteobacteria (52.31%–61.24%) persistently dominated the bacterial communities (Supplementary Fig. 2A). Thaumarchaeota (24.54%–78.06%) and Euryarchaeota (9.6%–43.00%) were the most abundant phyla in the archaeal communities (Supplementary Fig. 2B). Ascomycota (12.53%–43.28%) and Basidiomycota (7.2%–26.12%) dominated the fungal communities (Supplementary Fig. 2C). Stramenopiles (51.84%–80.91%) and Opisthokonta (11.02%–37.70%) were the major protists (Supplementary Fig. 2D).

**Figure 1:**
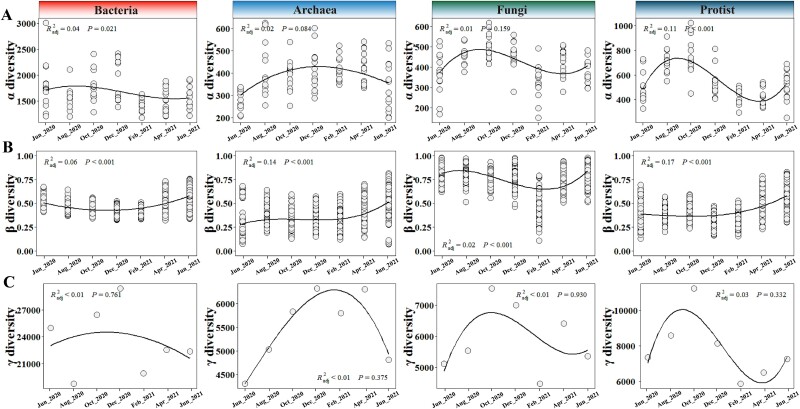
**Diversity patterns of intertidal microbial communities along a temporal gradient.** Linear fitting and polynomial regression methods were used to assess the relationship between (**A**) α-diversity (species richness), (**B**) β-diversity, and (**C**) γ-diversity and the sampling time. Four different microbial domains/kingdoms, including bacteria, archaea, fungi, and protists were analyzed.

Curvilinear patterns were observed for the seasonal dynamics of the α-, β-, and γ-diversity of intertidal microbiomes (Fig. 1A). The temporal dynamics of α-diversity (species richness) were generally similar across microbial domains except archaea, for which the richness peaked in June–August 2020, and bottomed in February–April 2021. For archaea, the temporal dynamics of α-diversity exhibited a unimodal pattern, with its zenith in October–December 2020. Similar temporal patterns could be observed for Shannon-Wiener diversity indices and Pielou evenness indices (Supplementary Fig. 3). The temporal dynamic of γ-diversity in general aligned with the changes observed in α-diversity across all investigated microbial domains (Fig. 1C). However, the patterns of β-diversity clearly diverged from those of α- and γ-diversity. Along the sampling timepoints, the shifts in β-diversity tended to exhibit unimodal patterns, whose valleys were found in December 2020–February 2021. Importantly, significant associations between microbial richness (α-diversity) and species pool size (γ-diversity) were identified (Supplementary Fig. 4). In contrast, significant associations between β-diversity and species pool size (γ-diversity) were not observable (Supplementary Fig. 5, A-D). Instead, notable correlations emerged between β-diversity and β-deviation indices (*bacteria: R^2^ = 0.27; archaea: R^2^ = 0.24; fungi: R^2^ = 0.25; protist: R^2^ = 0.10)* (*P < .001*) (Supplementary Fig. 5, E-H). This indicated that the temporal patterns of microbial β-diversity were likely to be influenced by the community assembly processes.

### Taxa-time and TDR

Two typical temporal patterns, including TTR and TDR, were investigated to determine the strength of seasonal turnovers of different microbial domains. First, TTR was assessed using a linear regression model and then plotted in a log–log space (Supplementary Fig. 6), the slopes of which reflect the degree of temporal turnover of intertidal microbiomes. Strong TTR patterns were observed for microbes of all four domains/kingdoms, of which fungi was the strongest and bacteria was the weakest (Supplementary Fig. 6). This indicated clearly differed living strategies along time for different microbial domains. Second, TDR was analyzed to investigate the strength of compositional variations along time for different microbial domains (Supplementary Fig. 7). Significant TDRs were only weakly found for fungi and protists, but not for bacteria and archaea, possibly due to the seasonal recovery of microbial composition over the sampling period (Supplementary Fig. 7). Such results suggested that fungi and protists could be more influenced by the environmental fluctuations along the temporal scale.

### Observed microbial β-diversity strongly deviated from null expectations

**Figure 2:**
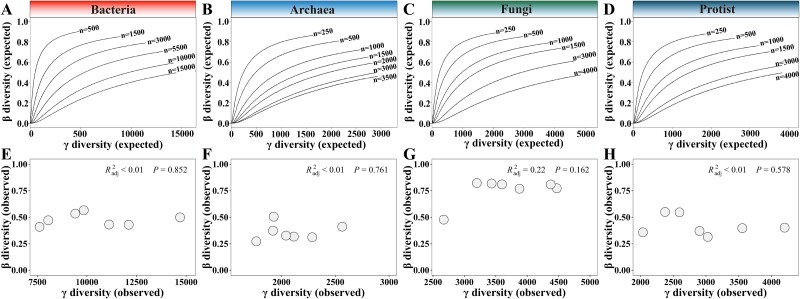
**The expected and observed relationships between β-diversity and γ-diversity for different microbial domains.** (**A**–**D**) the expected algebraic relationships between β- and γ-diversity for bacterial (A), archaeal (B), fungal (C), and protist communities (D) were simulated using random sampling, based on log-normal species abundance distributions. The curved relationship describes the trend of β diversity with γ diversity, and “n” represents the number of randomly sampled individuals. (**E**–**H**) The observed relationship between β-diversity and γ-diversity for different microbial domains.

To unravel the drivers underlying the seasonal fluctuations of microbial β-diversity, we first investigated whether the observed β-diversity was affected by γ-diversity. To do so, the algebraic relationship between γ-diversity and β-diversity were explored. Null model communities only encompassing random sampling were generated. The expected β-diversity increased with γ-diversity, and tended to saturate when γ-diversity further increased (Fig. 2, A-D). The number of individuals also affected β-diversity that small sampling size resulted in high β-diversity. However, clear associations were not found between the observed β-diversity and γ-diversity, regardless of which microbial domain (Fig. 2, E-H). This suggested that species pools had limited effects on the temporal variations of microbial β-diversity.

**Figure 3:**
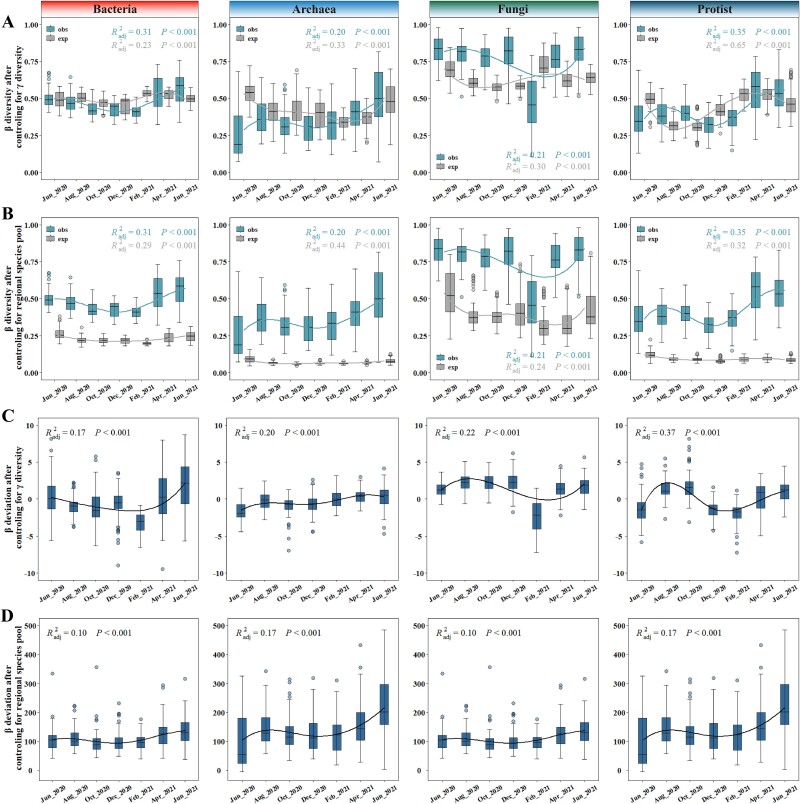
**Seasonal patterns of β-diversity and β-deviation.** (**A**) Seasonal patterns of observed (obs) and expected (exp) β-diversity for different microbial domains after controlling for γ diversity. (**B**) Seasonal patterns of observed (obs) and expected (exp) β-diversity for different microbial domains after controlling for regional species pools. (**C**) Dynamic patterns of β-deviations of different microbial domains after controlling for γ diversity. (**D**) Dynamic patterns of β-deviations of different microbial domains after controlling for regional species pools. (from left to right: bacterial community, archaeal community, fungal community, and protist community).

We subsequently investigated how the observed β-diversity deviated from null expectations when γ-diversity and temporal species pools were respectively controlled. Meanwhile, we also investigated the patterns of β-deviation, aiming to infer the drivers underlying the temporal dynamics of β-diversity. Remarkable disparities between the observed and expected β-diversity emerged under different controlling conditions. When γ-diversity was controlled, the expected β-diversity closely mirrored with the observed β-diversity (Fig. 3A). Notably, the expected β-diversity significantly deviated from the observed β-diversity in certain months, such as the ones for bacterial and archaeal communities from June 2020 to December 2020, and especially for fungal communities. When the temporal species pools were controlled, the expected β-diversity consistently deviated from the observed β-diversity with smaller values across all microbial domains (Fig. 3B), confirming that temporal species pools had limited effects on the compositional variations of microbial communities. Looking at β-deviations, similar temporal patterns were observed regardless which constraining approach was used for generating null models. When γ-diversity was controlled, the β-deviation values fluctuated near zero, indicating the importance of stochastic processes (Fig. 3C). When temporal species pools were controlled, the β-deviation values were generally above zero, suggesting the importance of heterogeneous selection and/or dispersal limitation (Fig. 3D). Comparatively, the above results suggested that community assembly processes, especially stochastic processes, may have strongly shaped the temporal dynamics of microbial β-diversity in the intertidal zone.

### Ecological processes shaping the temporal dynamics of intertidal microbiomes

**Figure 4:**
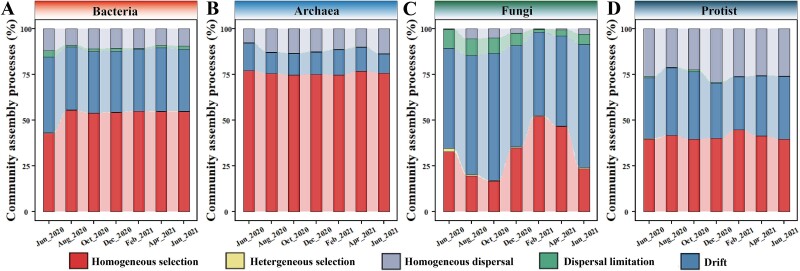
**Community assembly processes of intertidal microbial communities.** The contributions of different ecological processes to the composition of bacterial (A), archaeal (B), fungal (C), and protist (D) communities were quantified using a phylogenetic bin-based approach. Five different ecological processes were analyzed, including homogeneous selection, heterogeneous selection, drift, dispersal limitation, and homogeneous dispersal.

To quantify the contributions of community assembly processes to the temporal variations of intertidal microbiomes, a phylogenetic bin-based null model approach was employed. According to the above β-deviation analyses, the observed β-diversity was more similar to those of null models generated by controlling the γ-diversity (Fig. 3A). Therefore, controlling the γ-diversity was used as the constraints in generating null models. As a result, homogeneous selection and drift were the two major processes shaping the intertidal microbiomes. Different microbial domains were structured by different ecological processes (Fig. 4). Of these, bacteria were dominantly shaped by homogeneous selection and drift, archaea by homogeneous selection, fungi mainly by drift and homogeneous selection, and protists by homogeneous selection, drift, and homogeneous dispersal (Fig. 4). These suggested that different intertidal microbiomes were mediated by different community assembly processes.

### The relationship between microbial dynamics and environmental variables

**Figure 5:**
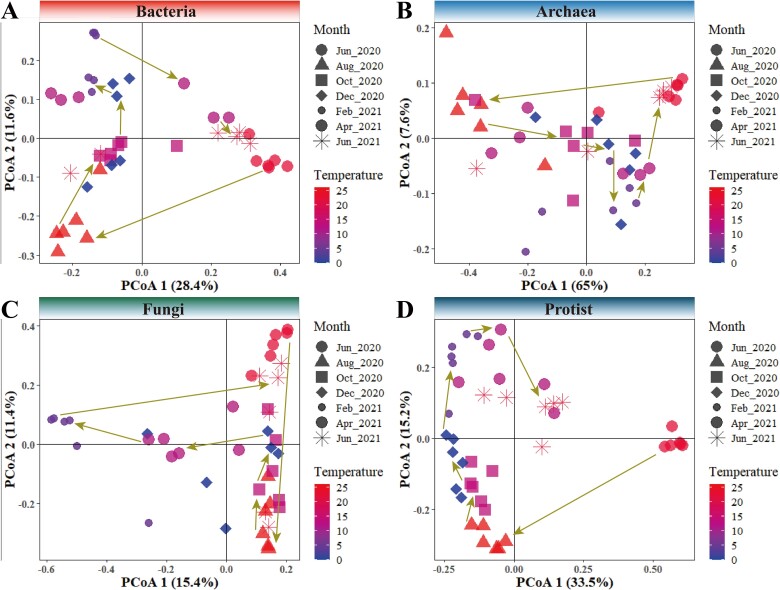
**Unconstrained PCoA showing the temporal dynamics of different microbial domains/kingdoms over the sampling months, including bacteria (A), archaea (B), fungi (C), and protists (D).** The Bray–Curtis dissimilarity was used to measure microbial compositional variations. Different shapes represent samples collected in different months. The temperature gradient is reflected in the change from low to high temperature. Each point on the graph represents a microbial community sample, and their distribution illustrates changes in community composition across sampling months and temperature gradients.

To investigate the relationship between microbial dynamics and environmental variables, we first investigated the compositional dynamics of intertidal microbiomes throughout the sampling period. Using PCoA, the compositional differences were viewed at a two-dimention space. The results showed clear differences in microbial compositions at different sampling month that samples collected at the same timepoints were tightly clustered. For all microbial domains, a trend of recovery in community composition could be observed, though the direction and routes greatly differed (Fig. 5). For instance, the community compositions of bacteria, archaea, and fungi in June 2021 were similar to those in June 2020, showing a pattern of returning. This suggested that seasonal factors such as temperature may have modulated the compositional variations of intertidal microbiomes.

We then investigated the environmental variables that may have shaped the compositional variations of intertidal microbiomes. Using partial Mantel test analyses, strong and significant correlations were observed between the compositional variations of intertidal microbiomes and environmental variables including temperature (fungi and protists) and pH (bacteria and protists) (Mantel’s *r* > 0.2, *P* < .01) (Supplementary Fig. 8A), suggesting that variations in temperature and pH may have exerted pronounced influences on the temporal dynamics of intertidal microbiomes. To further explore the impact of temperature on intertidal microbiomes, the relationship between community similarity and these two parameters were assessed using linear regression model (Supplementary Fig. 8B and C). The similarity of intertidal microbial composition significantly decreased with increasing temperature (*P < .001*), especially for fungi (*s* = −0.234, *R*2 adj = 0.20, *P* < .001), suggesting that increasement in temperature resulted in compositional variations of intertidal microbiomes. As another critical environmental factor, the increasement in pH significantly enhanced the similarity of bacterial (*s* = 1.44, *R*2 adj = 0.02, *P* < .001) and protist (*s* = 3.33, *R*2 adj = 0.04, *P* < .001) community compositions. These results confirmed the importance of temperature and pH in structuring the compositional variations of intertidal microbiomes.

## Discussion

Resolving the dynamic temporal patterns and the underlying driving mechanisms of microbial communities is one of the central topics in microbial ecology [34, 71]. In this study, comparative investigations were carried out across different microbial domains in a typical mudflat intertidal zone, aiming to reveal the underlying mechanisms driving the temporal dynamics of intertidal microbiomes.

### The temporal patterns differ across different intertidal microbial domains

Clear seasonal patterns were observed for the α- and γ-diversity of fungi and protists, but weakly or nonexistent for bacterial and archaeal communities (Fig. 1). Consistent with previous studies in different ecosystems [72–74], dynamic patterns in microbial β-diversity were observed across different microbial domains. However, only fungi showed a comparable temporal pattern in β-diversity to that of α- and γ-diveristy. This suggested that microbial β-diversity, though closely associated with α- and γ-diveristy [18], may be driven by multiple ecological processes [18, 19, 26, 29, 35, 75].

Community assembly processes primarily governed the temporal dynamics of β-diversity of intertidal microbial communities. Previously for plant communities, Kraft et al. demonstrate that sampling alone can predict the changes in β-diversity and there is no need to invoke local community assembly when explaining the global patterns of β-diversity [18]. In contrast, studies have also demonstrated the importance of local community assembly processes in shaping the β-diversity variation [19, 23, 74, 76, 77]. For microbial communities, local community assembly processes are found to play profound roles in shaping the variations of β-diversity [19, 74, 76]. Over the past years, a lot effort has been made to disentangle the relative importance of deterministic and stochastic processes in mediating microbial β-diversity variations [31, 78–81]. Importantly, the relative importance of local community assembly processes in driving β-diversity variations is scale-dependent, for both macrobial and microbial communities [20, 23]. Moreover, recent studies based on "saturation theory" have shown that mechanisms controlling microbial diversity can differ significantly depending on whether the microbiome is saturated or not [74]. In unsaturated communities, α diversity varies with the species pool, while β-diversity is primarily influenced by local community assembly processes. In saturated communities, both α diversity and β-diversity reached a stable upper limit and were no longer influenced by increasing species pool size. Consequently, local community assembly processes have a more significant impact on these saturated communities.

### Distinct processes mediated the temporal variations of intertidal microbiomes

To identify the drivers of the temporal dynamic patterns of microbial β-diversity, we assessed the relationships between β-diversity (observed and null expectations) and γ-diveristy [18, 19], extending the concept of regional species pool to temporal. No significant correlation was found, suggesting that temporal species pools had limited effects on the seasonal variations of microbial β-diversity. This aligns with a few recent studies showing that the spatial patterns in microbial β-diversity are strongly affected by local community assembly processes [19, 23, 75, 76].

Secondly, we analyzed the deviations of the observed β-diversity from null models [18, 19], to gain brief insights into how community assembly processes affected the compositional variations of intertidal microbiomes. Two approaches were employed to generate null model communities, either by controlling the γ-diversity or the regional species pool. These two types of null models respectively generated dissimilar and similar null communities, on the assumption that the microbial communities were respectively subjected to stochastic processes/heterogeneous selection and homogeneous selection [41]. Surprisingly here, the observed microbial β-diversity was more similar to that of null communities generated by controlling the γ-diversity, unlike in many studies that deviates from both [18, 26]. This suggested that stochastic processes may have dominated the compositional variations of intertidal microbiomes.

Temporal variation in β-diversity across different intertidal microbial domains may be driven by distinct ecological processes. To further investigate this, the contributions of different ecological processes were quantified using a phylogenetic bin-based null model approach [70]. Consistent with the β-deviation patterns, stochastic processes played important roles in structuring intertidal microbial composition, except for archaea. The archaeal communities were strongly structured by homogeneous selection, with the observed β-diversity significantly lower than null expectations in most sampling months. Different microbial domains differed greatly in the community assembly processes mediating compositional variations. Such differences could be due to the distinct physiological and biochemical traits carried by different microbial domains [82, 83]. However, this quite contrasts a recent study showing that small-sized bacteria are more influenced by dispersal-based stochastic processes, while large ones are more structured by selection-based deterministic processes [11]. Such inconsistency among different studies might be due to the different scales (e.g. global vs local) or dimensions (e.g. spatial vs temporal) undertaken [14, 35, 84, 85].

### Temperature acted as a major factor affecting intertidal microbiomes

Environmental conditions are also expected to influence microbial community assembly processes [86]. Multiple factors, such as pH and temperature, have been shown to primarily mediate the spatial patterns of microbial communities at large scales [87–89]. Interestingly, these two parameters were also found to be the major factors significantly associated with the temporal variations of intertidal microbiomes. PCoA analysis showed trends of seasonal recovery in compositions for almost all microbial domains, though the degree and routes of recovery varied. Similar patterns of recovery in microbial composition along recovered environmental conditions have also be observed in other ecosystems [90, 91], demonstrating that environmental conditions may exert strong influence on microbial community dynamics [86].

Here, we found that temperature tended to be the major factor mediating the temporal dynamics of intertidal microbiomes, and the fluctuation in temperature may have induced the fluctuation in microbial abundance, i.e. ecological drift, especially for fungi and protists. Considering various factors, microbial dormancy is expected to well explain such domain-level differences among prokaryotes (bacteria and archaea) and microeukaryotes (fungi and protists). Similar to that dormancy influences the patterns of microbial biogeography [92], the temporal patterns should also have been affected by dormancy. Specifically, bacteria and archaea are expected to combat and survive at the unfavorable temperature (low in winter and high in summer) via dormancy, whereas the majority of fungi (except spores) and protists perish or escape. This eventually results in high ecological drift for fungi and protists.

In conclusion, this study investigated the drivers of seasonal changes in the β-diversity across four microbial domains/kingdoms. We found that community assembly processes were mainly responsible for the temporal dynamics of intertidal microbiomes, and γ-diversity had limited effects on microbial β-diversity. Different ecological processes contributed differently to the assembly of microbial communities across domains. Among various environmental determinants, temperature and pH played crucial roles in shaping microbial temporal patterns, especially for fungi and protists. The results also indicated that microeukaryotes such as fungi and protists are less susceptible to environmental changes, and cautions shall be taken to maintain the stability of microeukaryotic communities for future ecosystem managements in the intertidal zones.

## Supplementary Material

Supplemental_Material_ycaf025

Supplementary_Table_1_ycaf025

## Data Availability

Sequencing data generated in this study are deposited at NCBI SRA portal under project ID PRJNA1029225.
