# Supplementary material for: Resolving ecological drivers of temporal variations of β-diversity across intertidal microbiomes
Source: ISME Commun. 2025 Feb 17;5(1):ycaf025. doi: 10.1093/ismeco/ycaf025 (PMC11879246; doi:10.1093/ismeco/ycaf025)
Supplement: Supplemental_Material_ycaf025 [file supplemental_material_ycaf025.pdf]

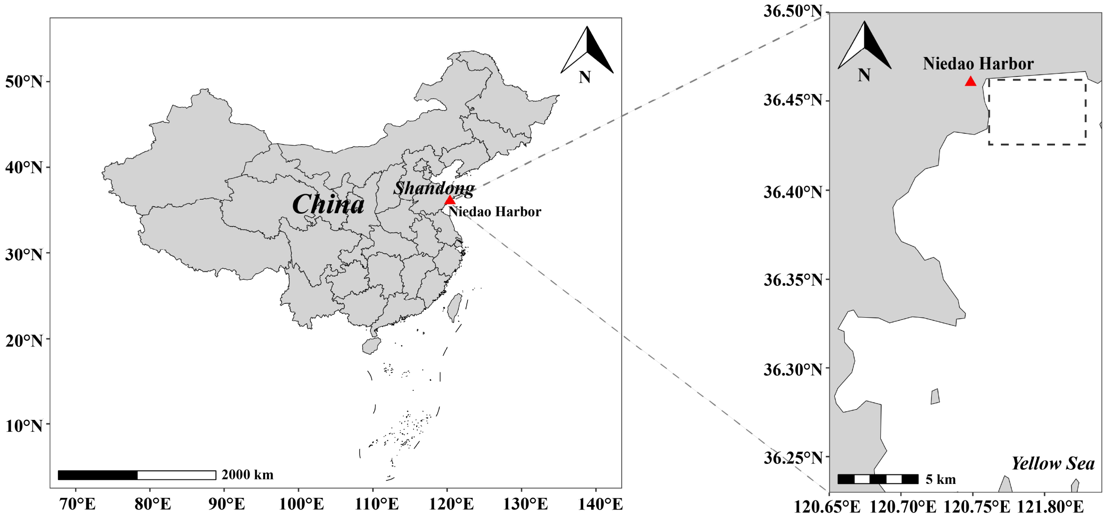

Supplementary Figure 1. The sampling site located near Niedao Harbor, a typical quagmire intertidal zone in Jimo District, Qingdao, China.

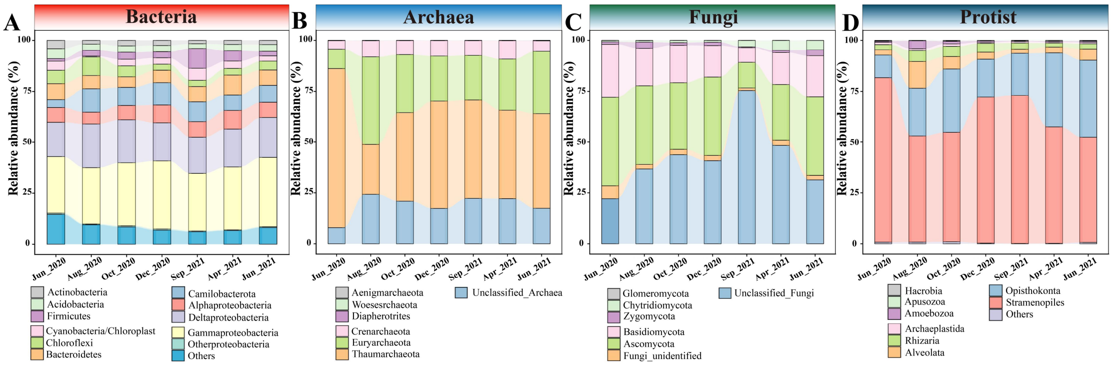

Supplementary Figure 2. Microbial community compositions at Phylum level. The diversity of **A**) Bacterial, **B**) Archaeal, **C**) Fungal, and **D**) Protista microbial communities along the temporal gradient were analyzed at the phylum level.

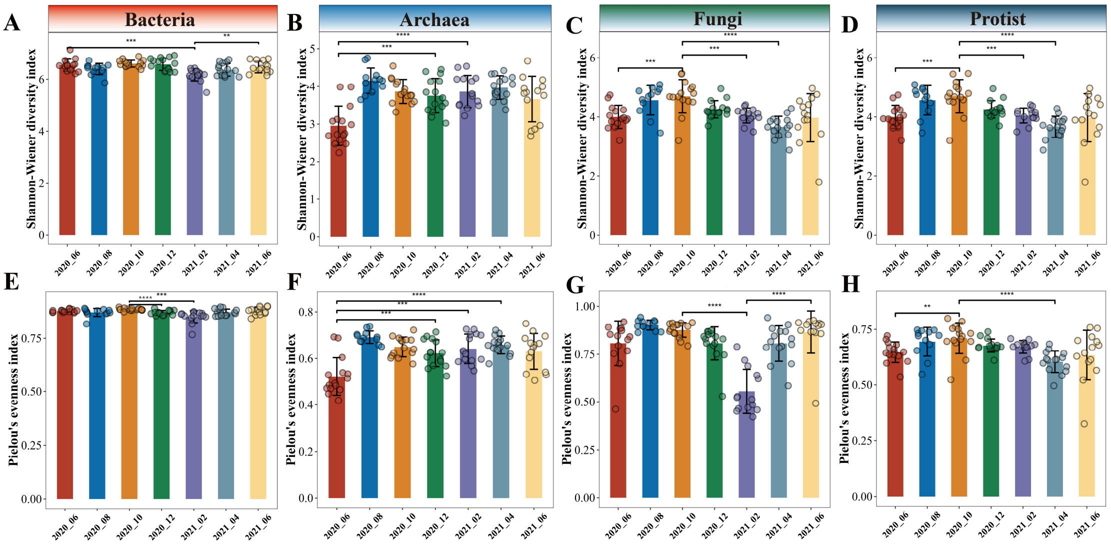

Supplementary Figure 3. The temporal dynamics of Shannon-Wiener diversity index and Pielou's evenness index for intertidal microbial communities. A) and E): Bacterial communities; B) and F): Archaeal communities; C) and G): Fungal communities; D)-H): Protist communities. The t-test was employed to compare the Shannon-Wiener diversity index and Pielou's evenness of microbial communities across various months in the intertidal zone.

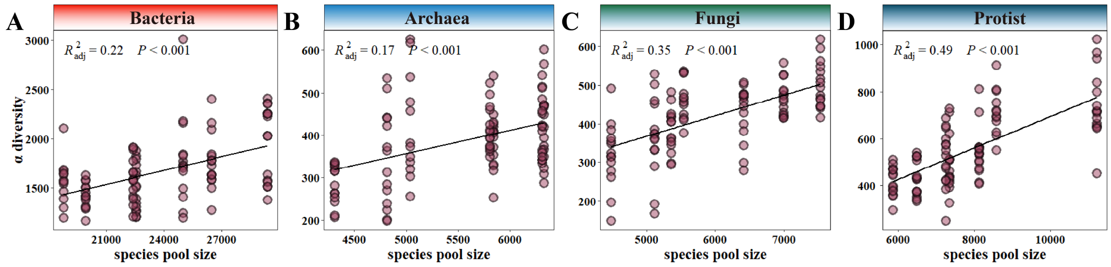

Supplementary Figure 4. Relationship between species pool size and α diversity of A) bacteria, B) archaea, C) fungi, and D) protists collected from samples across seven different successional stages in the intertidal zone. The α diversity is defined as the ASV richness of each sampling point.

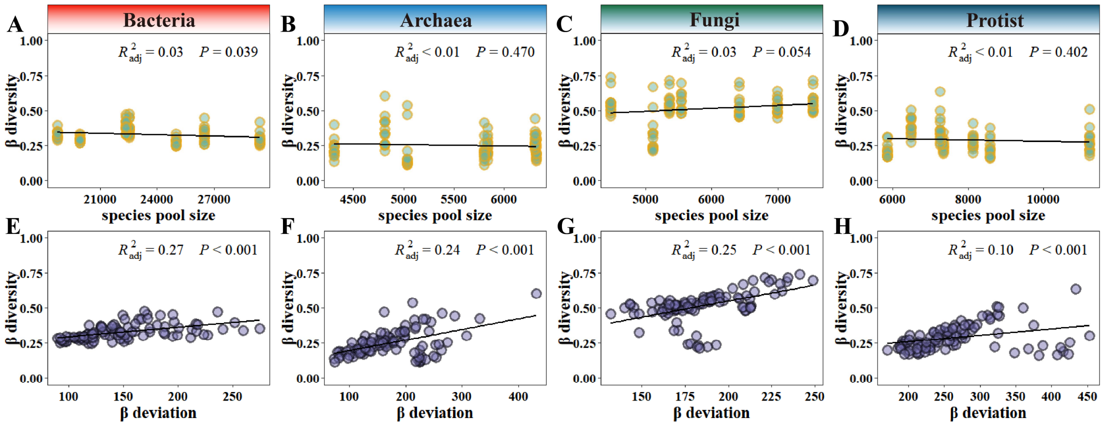

Supplementary Figure 5. A) to D) illustrate the relationship between β diversity and species pool size in intertidal microbial communities across various succession stages. Meanwhile, **E) to H)** depict the relationship between β diversity of intertidal microbial communities and the local community assembly process based on β deviation. Specifically, A) and E) correspond to the bacterial community, B) and F) to the fungal community, C) and G) to the archaeal community, and D) and H) to the protist community.

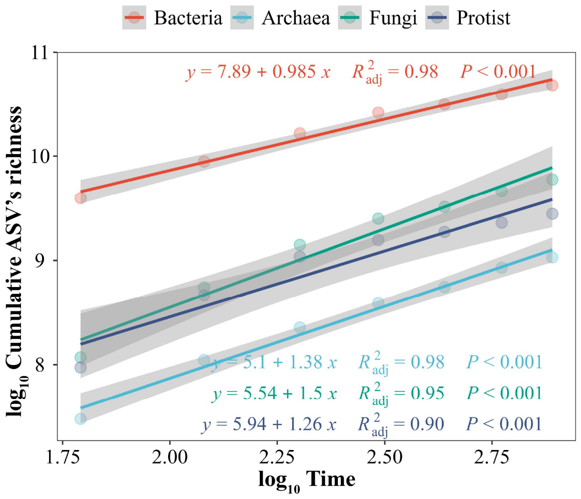

Supplementary Figure 6. Taxa-time relationship of intertidal microbial communities. The relationship between species richness and time is fitted using the power law equation $S = cT^{w}$, where S is the number of taxa observed, c is the constant, T is time and w is the index of the taxa-time relationship.

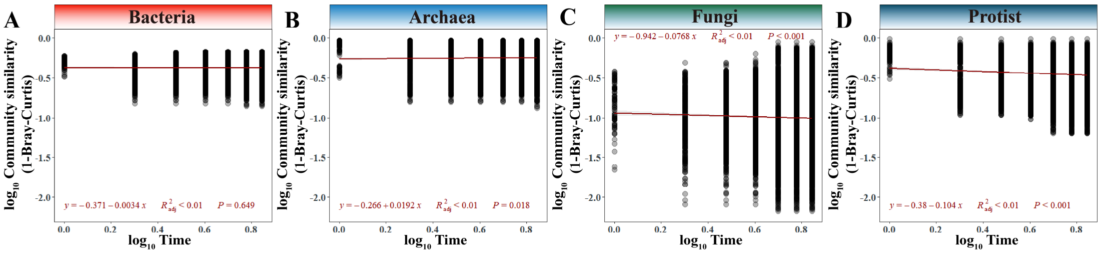

Supplementary Figure 7. Time-decay relationships (TDRs) of intertidal microbial communities. Linear regression assessed the relationship between temporal distance between samples and similarity in community composition of A) bacteria, B) archaea, C) fungi, and D) protists, respectively. The x-axis is the log-transformed cumulative time from the first to the last time point of the start of the sampling, and the y-axis is the log-transformed microbial community similarity index (based on Bray-Curtis).

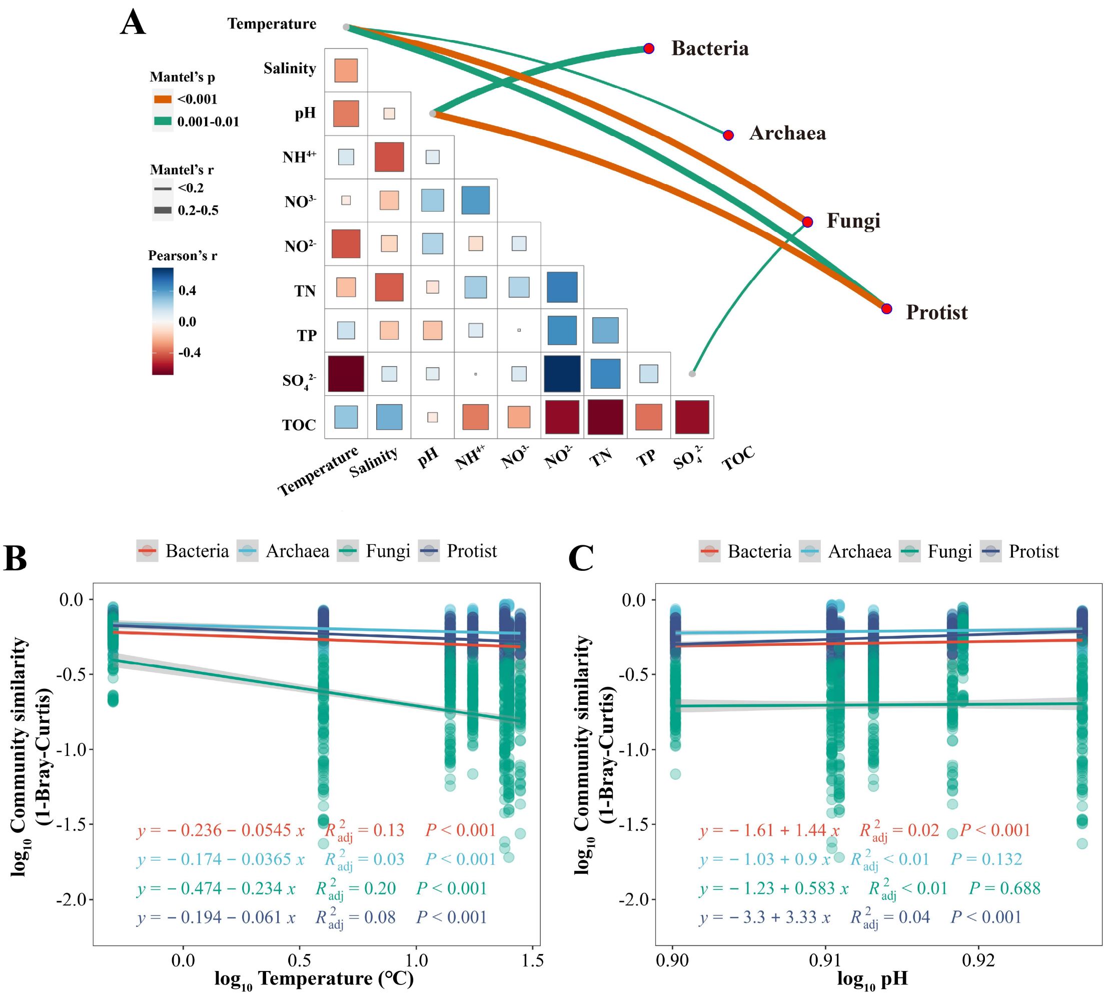

Supplementary Figure 8. The environmental driving factors of intertidal microbial community composition. A) Mantel tests were conducted to analyze the relationship between environmental variables and microbial community similarity. The Mantel test evaluates the strength and significance of correlation between the composition of bacterial, archaeal, fungal, and protist communities and the distance matrices of environmental variables by calculating Pearson correlation coefficient (r) and significance level (p-value). Intertidal microbial community similarity in relation to **B)** temperature and **C)** pH, respectively, with community similarity calculated as 1-Bray-Curtis dissimilarity.
