# Supplementary material for: Resolving ecological drivers of temporal variations of β-diversity across intertidal microbiomes
Source: ISME Commun. 2025 Feb 17;5(1):ycaf025. doi: 10.1093/ismeco/ycaf025 (PMC11879246; doi:10.1093/ismeco/ycaf025)
Supplement: Supplementary_Table_1_ycaf025 [file supplementary_table_1_ycaf025.docx]

**Supplementary Table 1.** Primer sequences used in Illumina amplicon sequencing and PCR assays

|  | **Primer name** | **Primer sequence (5’-3’)** | **Amplification region** | **Reference** |
| --- | --- | --- | --- | --- |
| **Bacteria** | 338F | CCTAYGGGRBGCASCAG | 16S rRNA V4 region | [1, 2] |
|  | 806R | GGACTACHVGGGTWTCTAAT |  |  |
| **Archaea** | 524F-10-ext | TGYCAGCCGCCGCGGTAA | 16S rRNA V4-V5 region | [3] |
|  | Arch958Rmod | CCGGCGTTGAVTCCAATT |  |  |
| **Fungi** | gITS-7F | GTGARTCATCGARTCTTTG | ITS2 region | [4] |
|  | ITS-4R | TCCTCCGCTTATTGATATGC |  |  |
| **protist** | 18S-528F | GCGGTAATTCCAGCTCCAA | 18S rRNA V4 region | [5] |
|  | 18S-706R | AATCCRAGAATTTCACCTCT |  |  |

**References**

1. Huse SM, Dethlefsen L, Huber JA *et al.* Exploring microbial diversity and taxonomy using ssu rrna hypervariable tag sequencing. *PLoS Genet*. 2008;**4**:e1000255 <https://doi.org/10.1371/journal.pgen.1000255>

2. Caporaso JG, Lauber CL, Walters WA *et al.* Global patterns of 16s rrna diversity at a depth of millions of sequences per sample. *Proceedings of the National Academy of Sciences*. 2011;**108 Suppl 1**:4516-22 <https://doi.org/10.1073/pnas.1000080107>

3. Pires AC, Cleary DF, Almeida A *et al.* Denaturing gradient gel electrophoresis and barcoded pyrosequencing reveal unprecedented archaeal diversity in mangrove sediment and rhizosphere samples. *Applied and Environmental Microbiology*. 2012;**78**:5520-8 <https://doi.org/10.1128/AEM.00386-12>

4. McGuire KL, Payne SG, Palmer MI *et al.* Digging the new york city skyline: Soil fungal communities in green roofs and city parks. *PLoS One*. 2013;**8**:e58020 <https://doi.org/10.1371/journal.pone.0058020>

5. Cheung MK, Au CH, Chu KH *et al.* Composition and genetic diversity of picoeukaryotes in subtropical coastal waters as revealed by 454 pyrosequencing. *The ISME Journal*. 2010;**4**:1053-9 <https://doi.org/10.1038/ismej.2010.26>
